# Primary Chest Wall Malignant Lymphoma in a Patient Without a History of Tuberculosis or Chronic Pyothorax: A Case Report With a Review of the Literature

**DOI:** 10.7759/cureus.114033

**Published:** 2026-08-05

**Authors:** Toru Niitsuma, Yoshihisa Saida, Takaharu Kiribayashi, Osahiko Hagiwara, Kazuki Ito

**Affiliations:** 1 Department of Surgery, Toho University Ohashi Medical Center, Tokyo, JPN

**Keywords:** case report, chest wall tumor, dlbcl, malignant lymphoma, rib fracture

## Abstract

Primary malignant lymphoma of the chest wall is a rare disease. Its pathogenesis has been reported to be associated with chronic pyothorax following tuberculous inflammation, artificial pneumothorax, and Epstein-Barr virus (EBV) infection. We herein report a surgically treated case of primary chest wall lymphoma without a history of tuberculosis or chronic pyothorax. A 65-year-old woman with a history of left rib fractures caused by domestic violence was found to have an abnormal shadow in the left chest on a chest radiograph obtained during a routine medical checkup. Contrast-enhanced computed tomography (CECT) revealed a mass lesion in the left chest wall, and she was referred to our department for further evaluation and treatment. Examination demonstrated a gradually enlarging mass measuring 30 mm × 10 mm located just below the left seventh rib. No obvious invasion into the ribs or surrounding organs and tissues was observed. Tumor resection was performed under thoracoscopic and direct visualization. Histopathological examination revealed diffuse large B-cell lymphoma (DLBCL). We experienced a surgically treated case of primary chest wall malignant lymphoma without any apparent underlying disease that could have contributed to its development.

## Introduction

Primary malignant lymphoma of the chest wall is a rare clinical entity that accounts for only a small proportion of extranodal lymphomas [1]. Most reported cases are classified as pyothorax-associated lymphoma (PAL), a subtype of non-Hodgkin lymphoma, most commonly diffuse large B-cell lymphoma (DLBCL), that develops in the pleural cavity after longstanding chronic pyothorax caused by tuberculous pleuritis or artificial pneumothorax therapy for pulmonary tuberculosis [2-5]. According to the World Health Organization classification, PAL develops in approximately 2% of patients 22-55 years after the onset of tuberculosis [2]. Epstein-Barr virus (EBV) infection has been implicated in the pathogenesis of PAL through persistent chronic inflammation and local immunosuppression [5-7]. In contrast, primary chest wall lymphoma occurring in patients without a history of tuberculosis, chronic pyothorax, or other predisposing inflammatory conditions is exceedingly rare, and its clinical presentation and radiological findings are often nonspecific, making preoperative diagnosis challenging [8-10]. We herein present a surgically treated case of primary chest wall lymphoma in a patient with no history of tuberculosis or chronic pyothorax and discuss its clinical features with a review of the relevant literature.

## Case presentation

History of present illness

**Figure 1:**
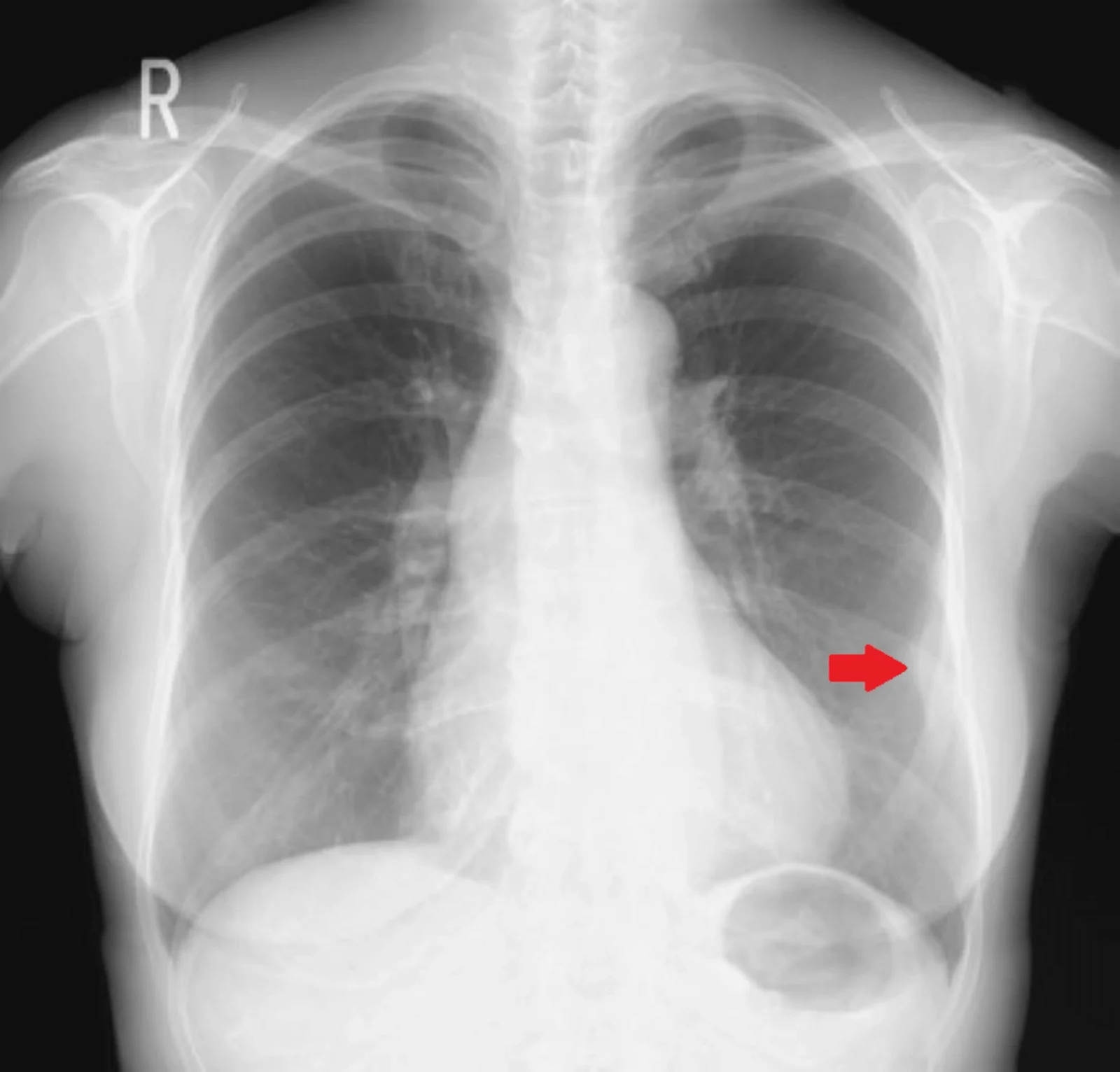
Chest radiograph Chest radiograph demonstrating a nodular shadow (red arrow) projecting over the left lower lung field along the inner border of the left seventh rib.

A 65-year-old woman was referred to our department for evaluation of a left chest wall mass detected during a routine health screening. Her medical history was significant for hypertension, a previous left rib fracture resulting from intimate partner violence, *Mycoplasma pneumonia*, and herpes zoster. She had no history of tuberculosis, chronic pyothorax, or anti-tuberculosis treatment. Her family history was unremarkable. In January 2025, a chest radiograph obtained during a routine medical checkup revealed an abnormal shadow in the left lower thorax. Subsequent contrast-enhanced computed tomography (CECT) performed in February demonstrated a chest wall mass, and she was referred to our institution in April 2025 for further evaluation and treatment. She was asymptomatic except for mild spontaneous tingling pain extending from the left subcostal region to the left shoulder. She denied fever, cough, weight loss, or other constitutional symptoms. She had never smoked and had no history of asbestos exposure. On admission, the patient's vital signs were stable, with a body temperature of 36.6°C, blood pressure of 117/65 mmHg, and a regular pulse rate of 58 beats/min. Cardiopulmonary examination was unremarkable, and no superficial lymphadenopathy was detected. The patient complained of spontaneous tingling pain extending from the left subcostal region to the left shoulder. Laboratory investigations, including a complete blood count and serum biochemical tests, were within normal limits. Chest radiography demonstrated a nodular shadow in the left lower lung field (Figure 1).

**Figure 2:**
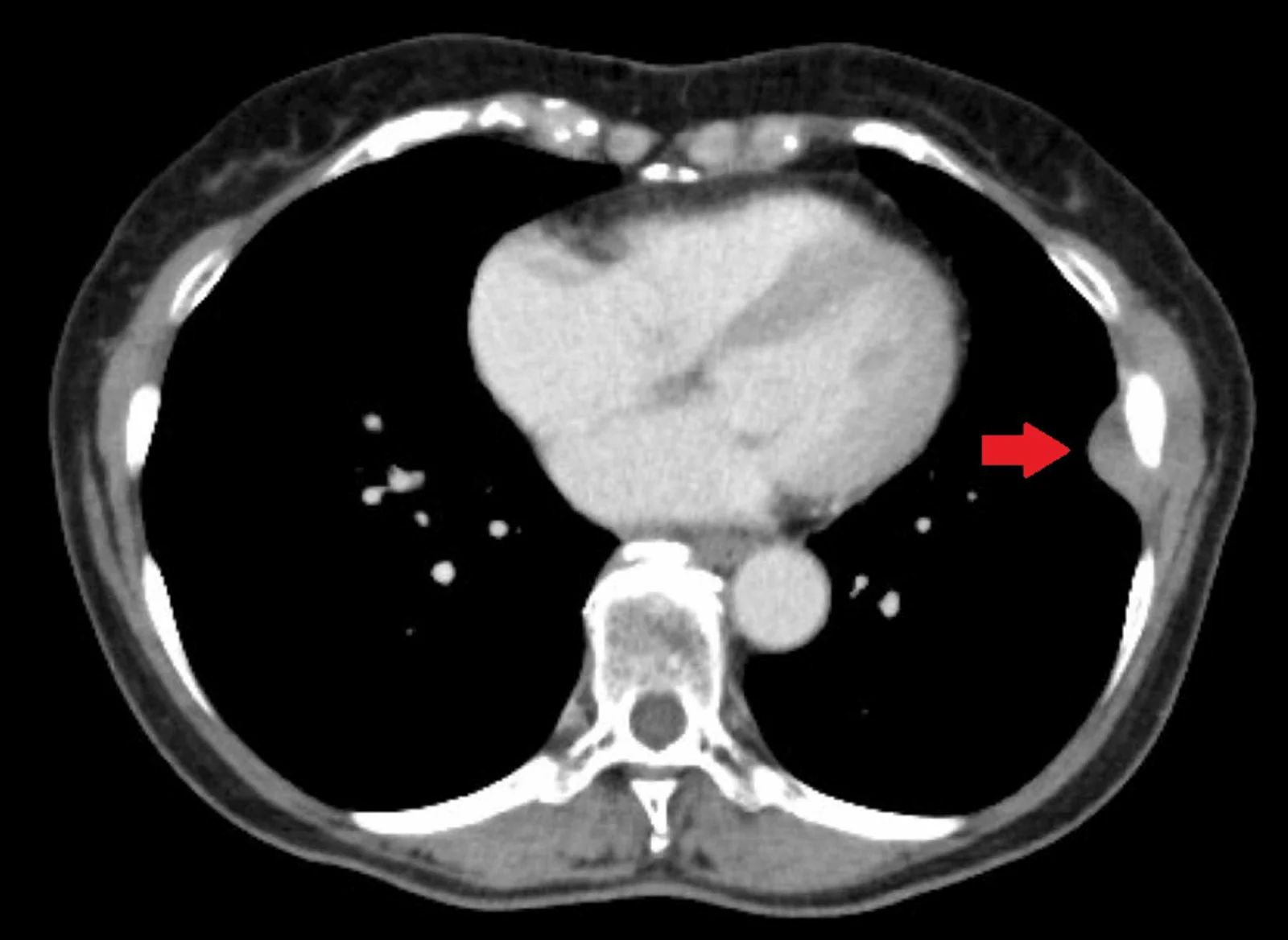
CECT CECT revealed a 30 mm × 10 mm chest wall mass located just below the left seventh rib. CECT: contrast-enhanced computed tomography

Contrast-enhanced CT revealed a well-defined 30 mm × 10 mm soft-tissue mass located beneath the left seventh rib (Figure 2).

The lesion exhibited no significant contrast enhancement. There was no evidence of invasion of the adjacent rib, lung, or surrounding structures. No hilar or mediastinal lymphadenopathy was identified, and no distant metastatic lesions were detected. Based on the radiological findings, including the absence of rib invasion, lymphadenopathy, or distant metastases, a benign chest wall tumor or post-traumatic chest wall change associated with the previous rib fracture was considered more likely than malignant lymphoma. After discussing management options with the patient, surgical resection was performed in June 2025 for definitive diagnosis and treatment.

Operative findings

**Figure 3:**
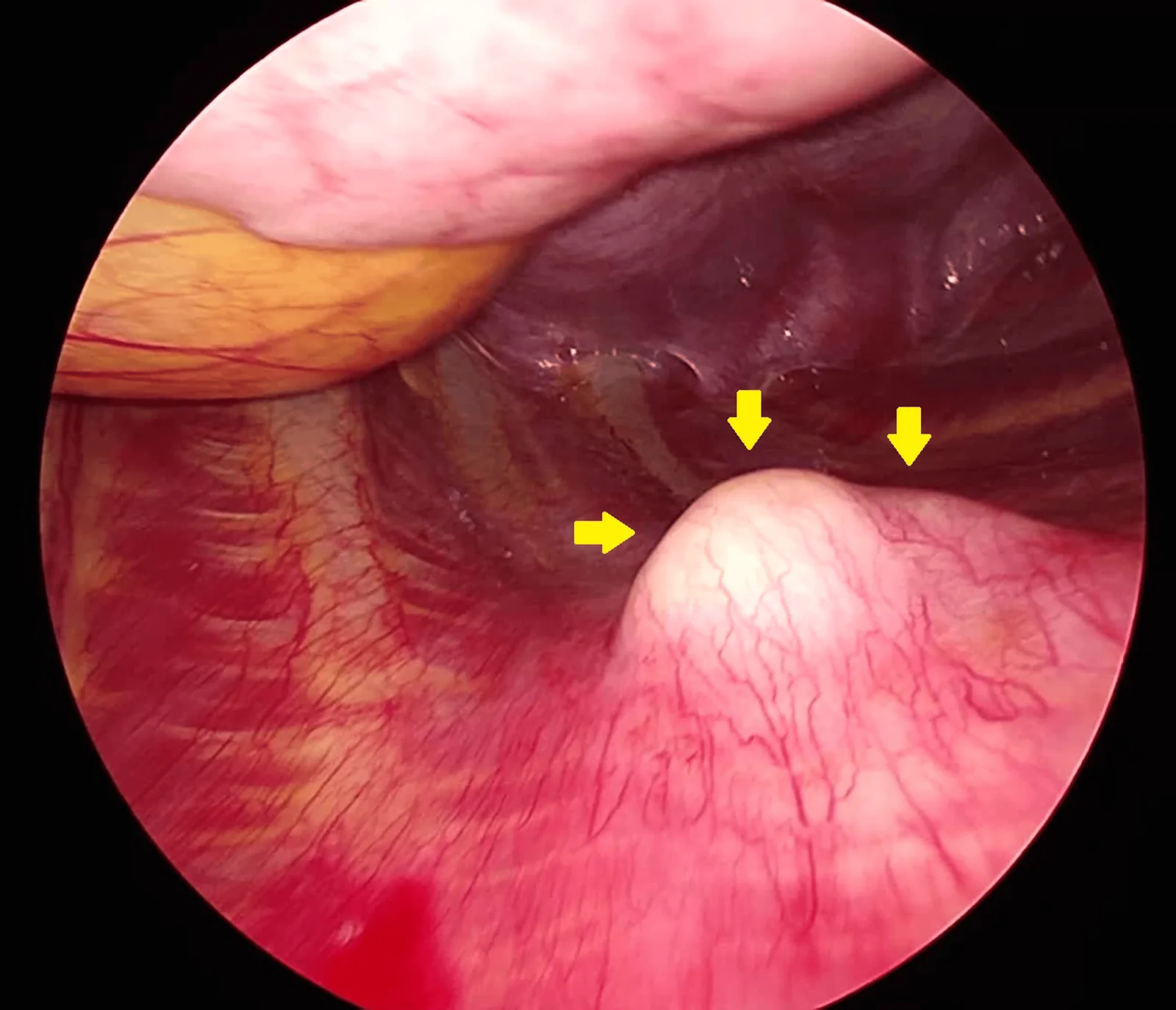
Operative findings Thoracoscopic examination revealed a localized bulging lesion of the chest wall corresponding to the known tumor beneath the seventh rib along the midaxillary line.

The operation was performed under general anesthesia with epidural analgesia. One-lung ventilation was achieved using a left-sided double-lumen endotracheal tube (35 Fr). The patient was placed in the right lateral decubitus position, and three-port video-assisted thoracoscopic surgery (VATS) was initiated. Thoracoscopic examination revealed a localized bulging lesion of the chest wall corresponding to the known tumor beneath the seventh intercostal space along the midaxillary line (Figure 3).

No pleural effusion or pleural adhesions were present. The lesion was initially dissected thoracoscopically with an intended surgical margin of at least 2 cm. However, intraoperative findings demonstrated that the tumor extended beyond the seventh intercostal space into the subcutaneous tissue overlying the seventh rib, making complete resection through a thoracoscopic approach alone difficult. After circumferential mobilization of the lesion from the thoracic cavity, a 3 cm skin incision was made directly over the tumor. Through the additional extrathoracic approach, the seventh rib was exposed, and the tumor was found to extend through the seventh intercostal muscle, partially covering the surface of the rib. Careful dissection confirmed the absence of direct rib invasion. The tumor was subsequently resected en bloc with adequate surgical margins.

Pathological findings

**Figure 4:**
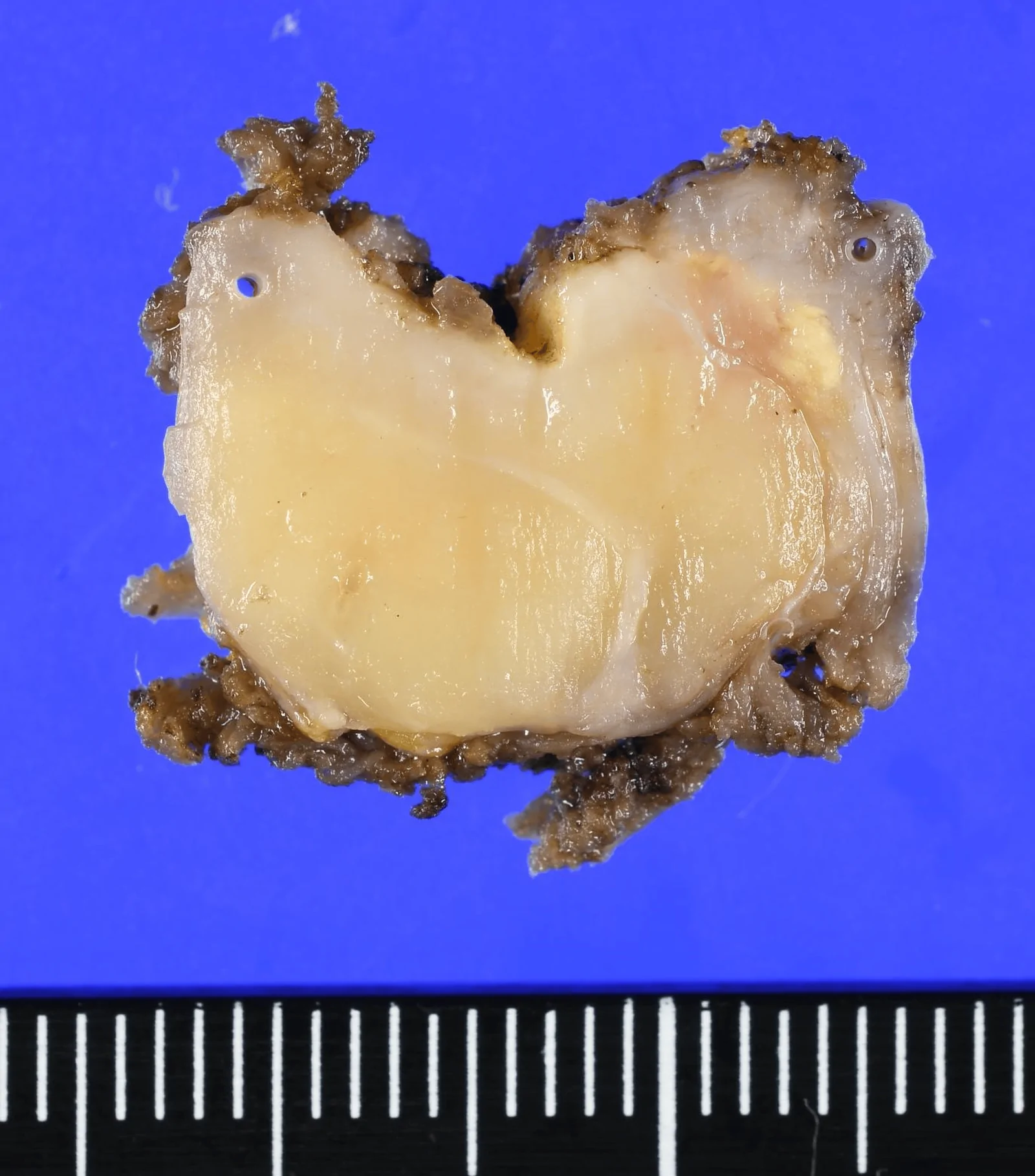
Cut surface of the resected tumor The resected specimen showed a solid white tumor measuring 45 mm × 40 mm × 10 mm with focal necrosis on the cut surface.

Gross examination revealed a solid white tumor measuring 45 mm × 40 mm × 10 mm with focal necrosis on the cut surface (Figure 4).

**Figure 5:**
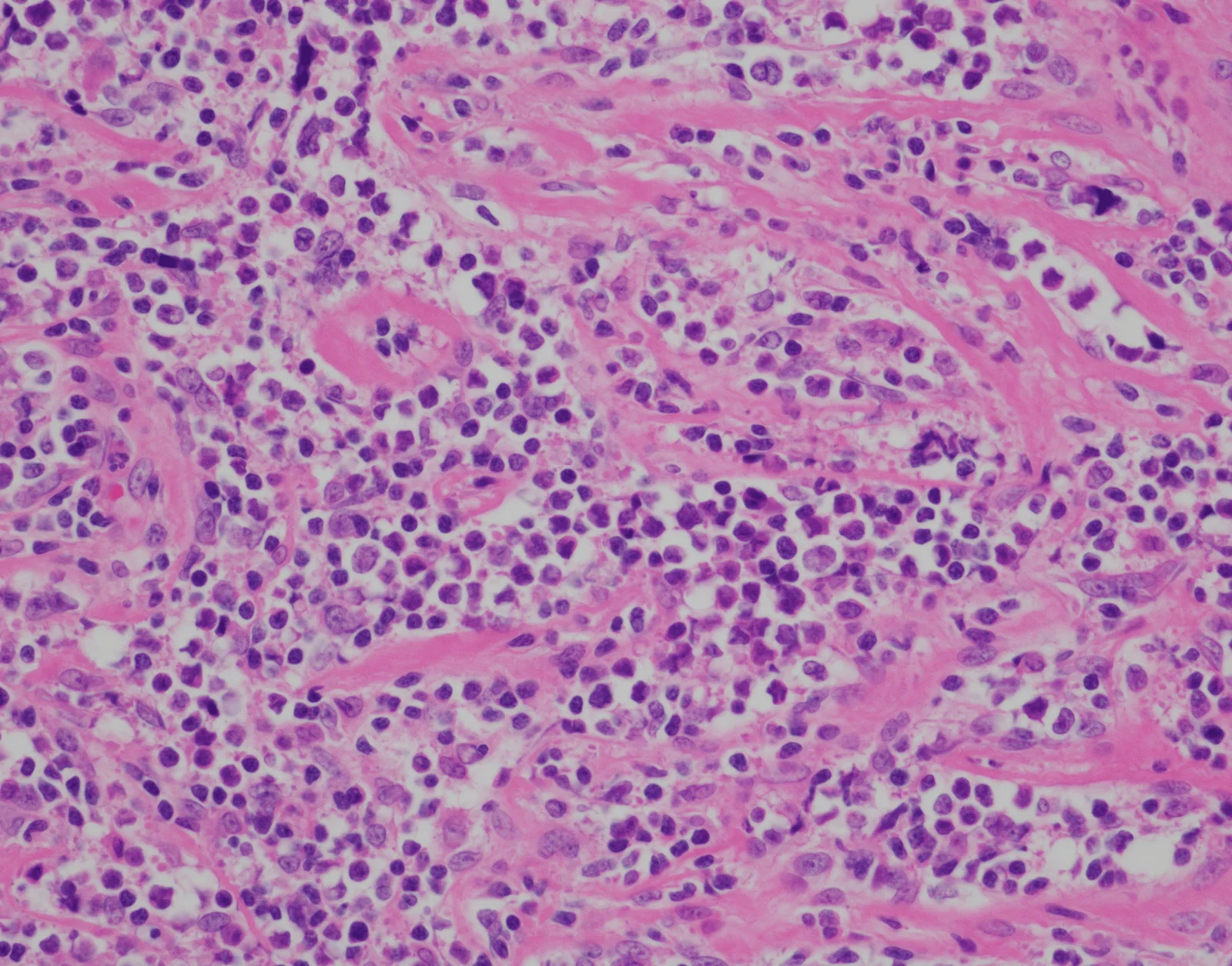
H&E staining, ×20 magnification The tumor showed diffuse proliferation of atypical lymphoid cells within a fibrous stroma. The neoplastic cells were small to medium in size with hyperchromatic nuclei and irregular nuclear contours. H&E: hematoxylin and eosin

Microscopically, diffuse proliferation of atypical lymphoid cells was observed within a fibrous stroma. The tumor cells were small to medium in size and exhibited hyperchromatic nuclei with irregular nuclear contours (Figure 5).

**Figure 6:**
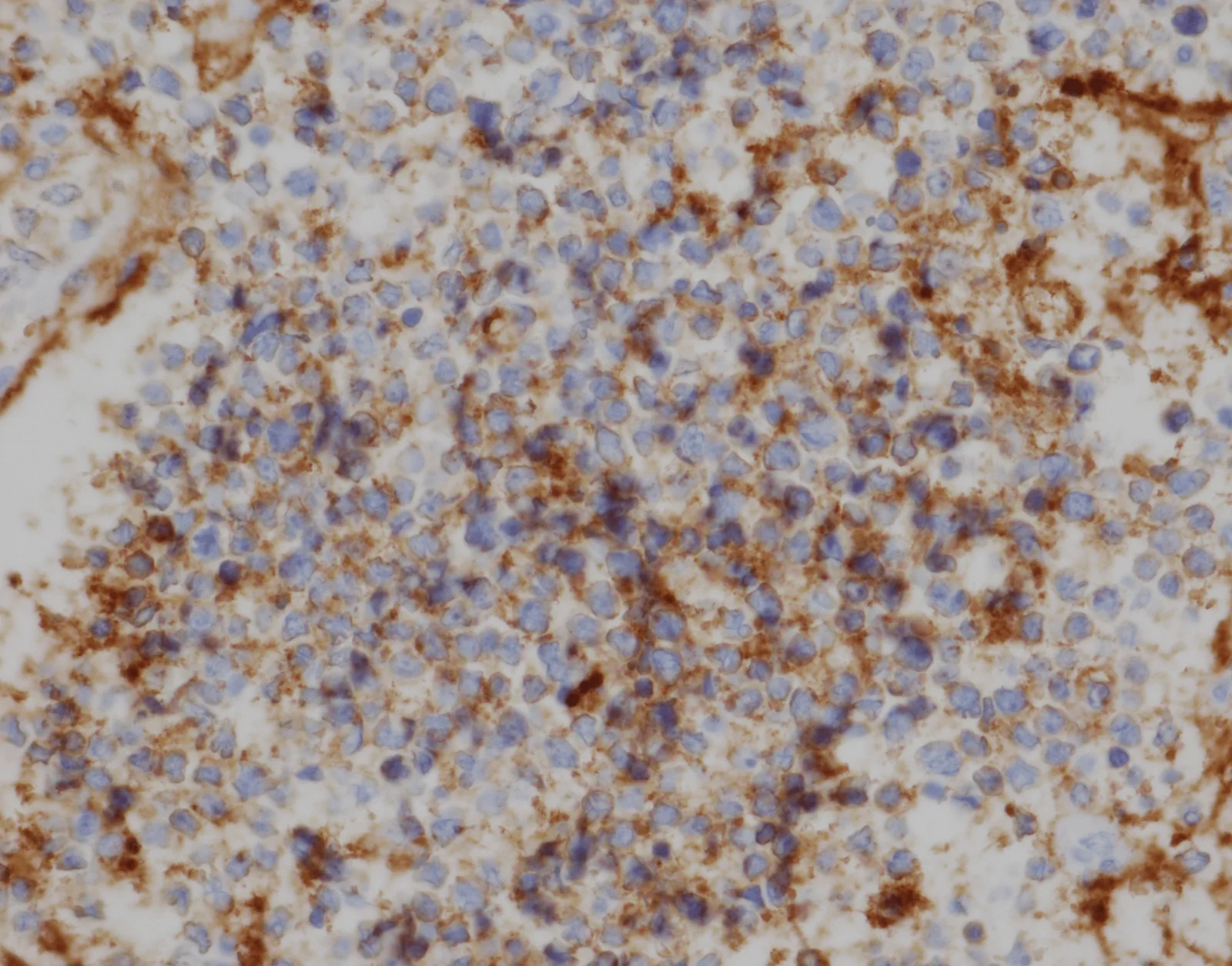
Immunohistochemical staining for CD10 Tumor cells showed strong membranous positivity for CD10, indicating a germinal center B-cell phenotype. CD10: cluster of differentiation 10

**Figure 7:**
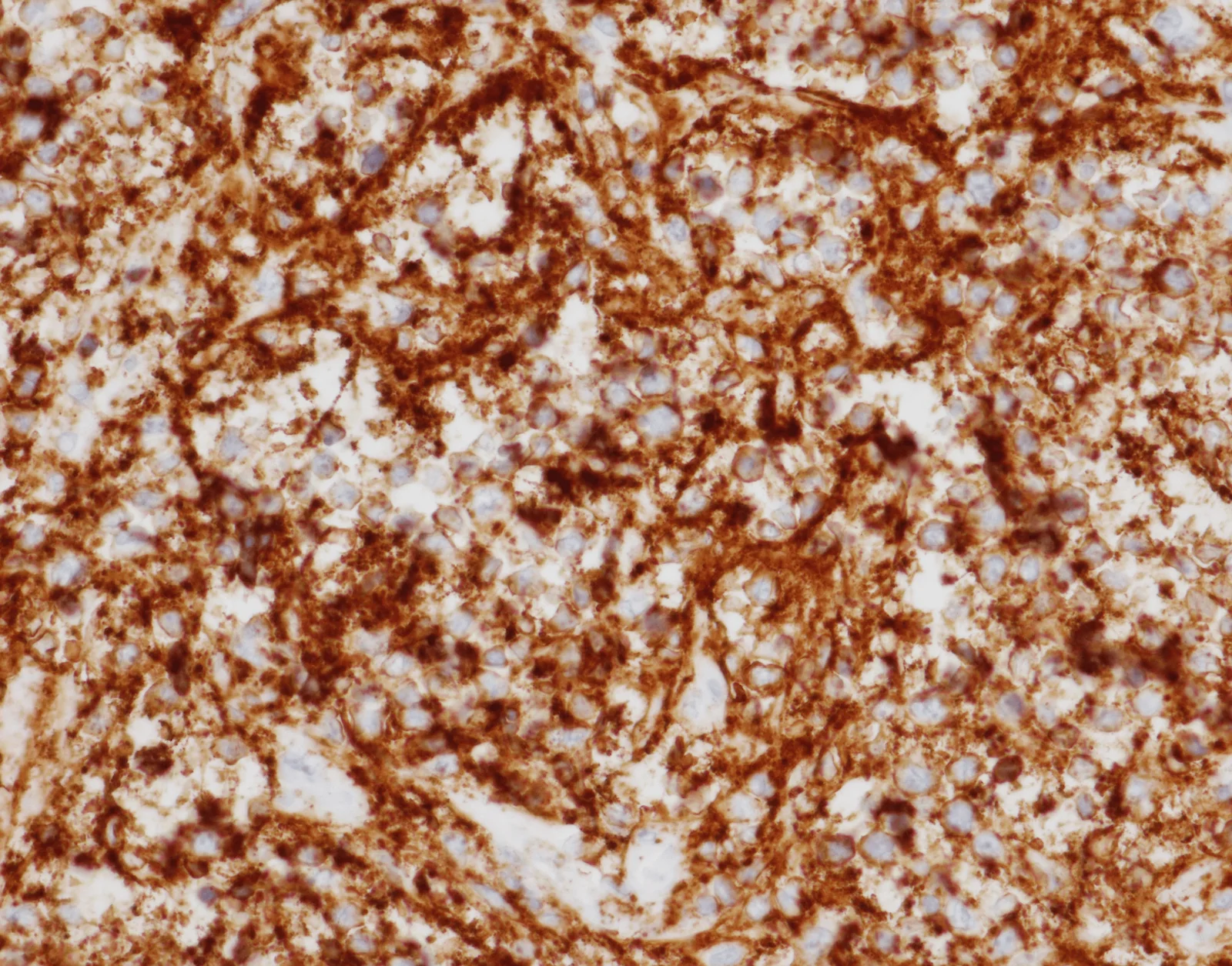
Immunohistochemical staining for CD20 Tumor cells demonstrated diffuse strong positivity for CD20, confirming B-cell lineage. CD20: cluster of differentiation 20

Immunohistochemical analysis demonstrated strong positivity for CD10 (cluster of differentiation 10) (Figure 6), and the cells were also positive for CD20 (cluster of differentiation 20) (Figure 7).

Based on these findings, the tumor was diagnosed as DLBCL.

Postoperative course

**Figure 8:**
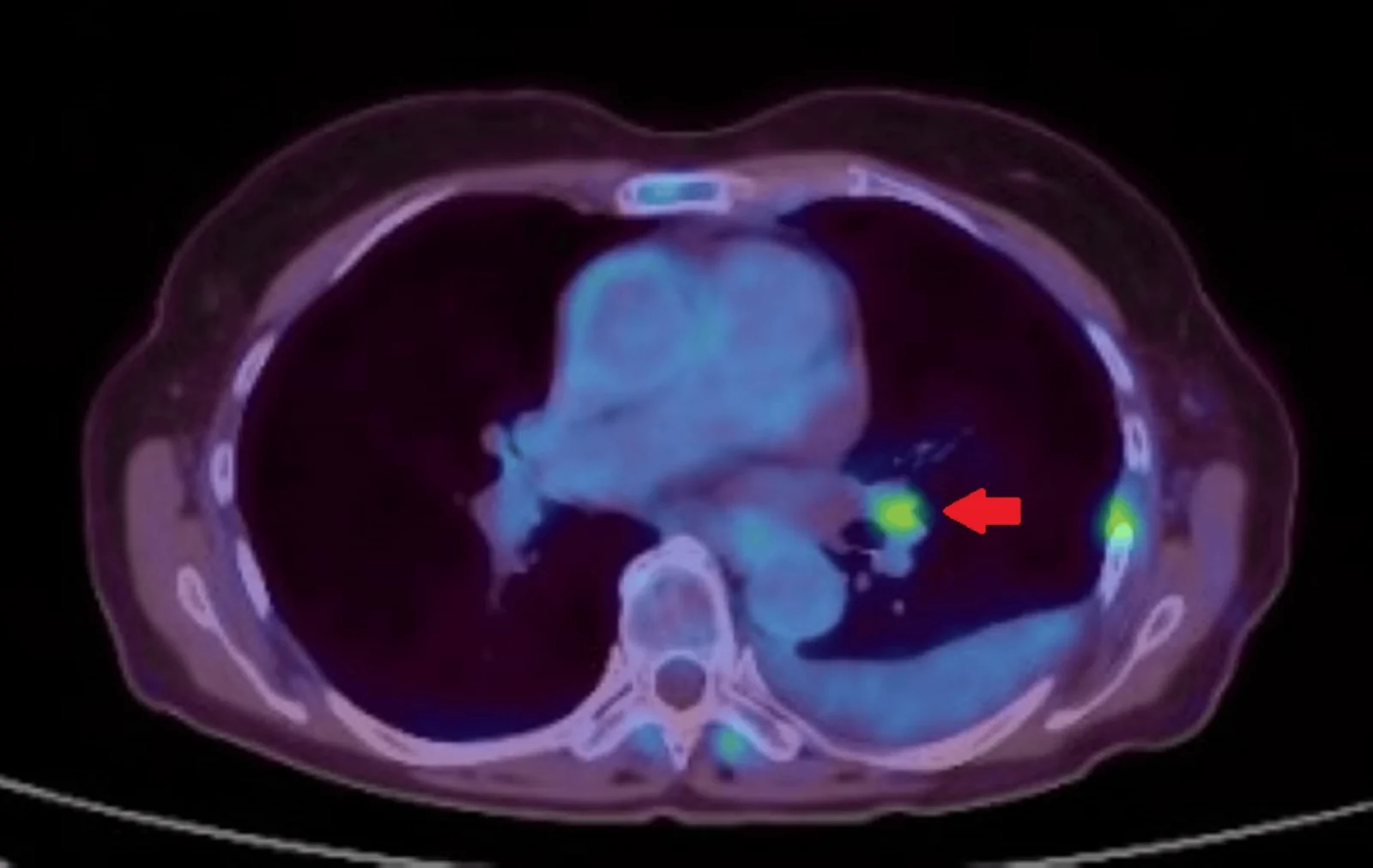
FDG chest PET-CT findings FDG PET-CT showing increased FDG uptake in the left mediastinal lymph node. The findings were suggestive of lymphomatous lymph node involvement. FDG: fluorodeoxyglucose; PET-CT: positron emission tomography-computed tomography

**Figure 9:**
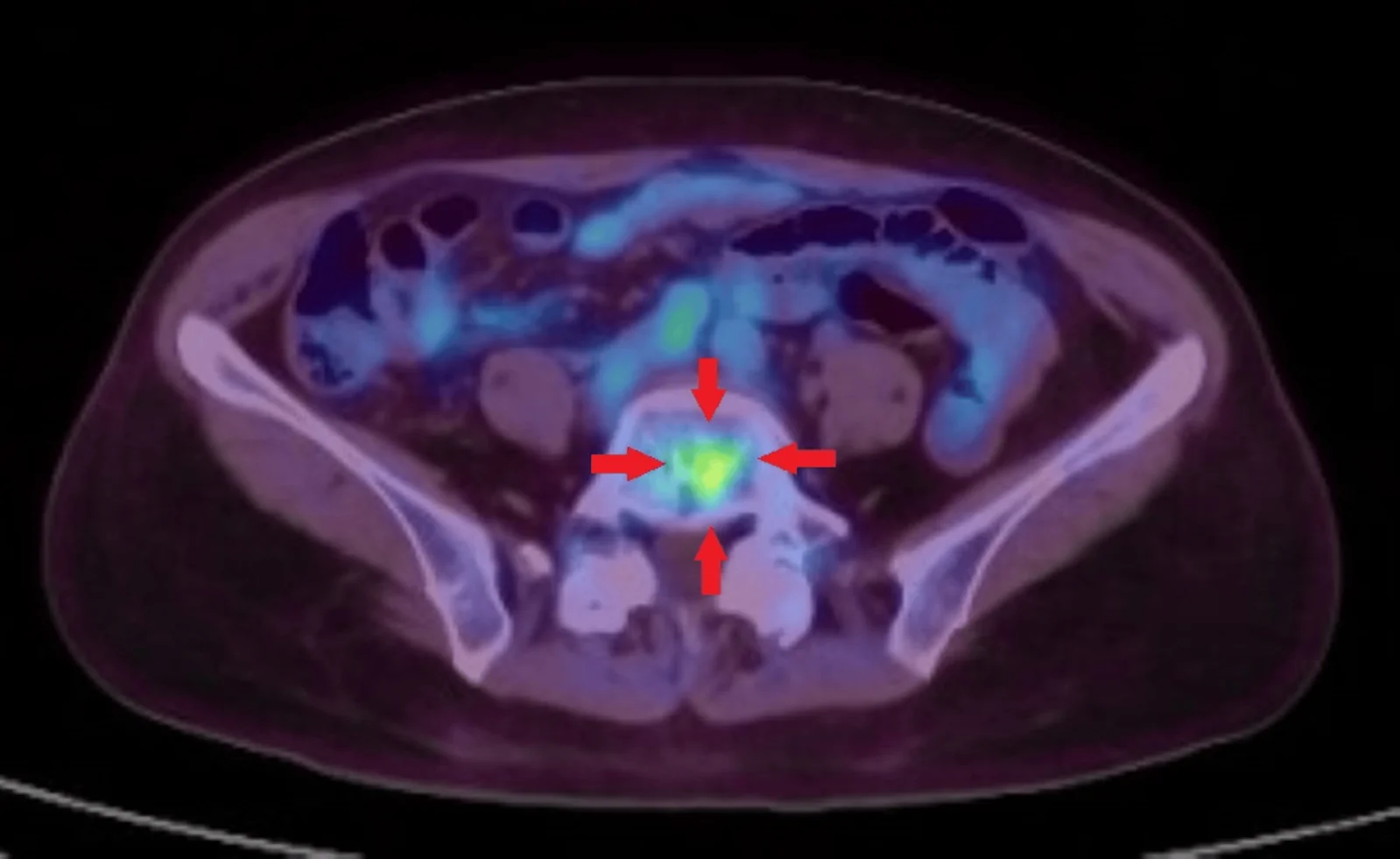
FDG PET-CT findings of the fifth lumbar vertebra FDG PET-CT showing increased FDG uptake in the fifth lumbar vertebra. The findings were suggestive of possible osseous involvement by lymphoma. FDG: fluorodeoxyglucose; PET-CT: positron emission tomography-computed tomography

The postoperative course was uneventful. The chest tube was removed on postoperative day 2, and the patient was discharged on postoperative day 4. Following the pathological diagnosis, fluorodeoxyglucose (FDG) positron emission tomography-computed tomography (PET-CT) was performed for systemic staging. PET-CT demonstrated increased FDG uptake in the bilateral hilar and mediastinal lymph nodes, including the left mediastinal lymph node (Figure 8). It also demonstrated increased FDG uptake in the third and fifth lumbar vertebrae (Figure 9), suggesting possible osseous involvement by lymphoma.

These findings suggested primary chest wall DLBCL with suspected hilar and mediastinal lymph node involvement and possible osseous involvement of the lumbar spine. However, because pathological confirmation of these lesions was not obtained, the precise extent of disease and Ann Arbor stage could not be determined. The patient was subsequently referred to a tertiary referral center with a hematology service for further treatment. Detailed information regarding subsequent chemotherapy, treatment response, and long-term clinical outcomes after referral was unavailable at the time of manuscript preparation.

## Discussion

We report a rare case of primary chest wall DLBCL occurring in a patient without a history of chronic inflammatory conditions. Primary non-Hodgkin lymphoma arising from the pleura or chest wall is uncommon, accounting for only 0.3%-1% of all extranodal lymphomas [1]. Most reported cases are B-cell lymphomas associated with tuberculous pleuritis or chronic pyothorax [3,4].

Clinically, pleural and chest wall lymphomas typically occur in middle-aged to elderly men with a history of tuberculosis or chronic pyothorax. Chronic local inflammation has been proposed as a major pathogenic factor, as persistent antigenic stimulation may promote B-cell proliferation and subsequent lymphomagenesis [3,4]. In addition, an association with EBV infection has been reported [5,6]. Other potential risk factors include cytokine-mediated inflammation, particularly involving interleukin-6, previous radiation exposure, autoimmune disorders, immunosuppressive therapy, and immunodeficiency states such as human immunodeficiency virus (HIV) infection.

The present patient had none of these recognized risk factors and did not fit the typical clinical profile of a middle-aged or elderly man. Because she had previously sustained a left rib fracture secondary to intimate partner violence, post-traumatic chest wall changes were initially considered in the differential diagnosis. However, postoperative pathological examination revealed DLBCL, and subsequent FDG PET-CT demonstrated suspected hilar and mediastinal lymph node involvement, as well as areas of increased FDG uptake in the vertebrae suggestive of possible osseous involvement. Had the lesion been regarded solely as a post-traumatic change and managed conservatively with radiological follow-up, further disease progression might have occurred before a definitive diagnosis was established.

To our knowledge, no previous cases of primary chest wall lymphoma developing after a rib fracture have been reported in Japan, and the relationship between these conditions remains unclear. In PAL, the interval between artificial pneumothorax treatment and lymphoma diagnosis is typically prolonged, averaging 30-40 years [8]. Therefore, it appears unlikely that a relatively short-term tissue injury such as a rib fracture directly contributed to lymphoma development in the present case. Nevertheless, a previous report described chest wall lymphoma arising 19 years after lung cancer surgery within a postoperative residual cavity associated with chronic inflammation [9]. This observation suggests that chronic inflammatory changes induced by previous physical injury or surgical intervention may theoretically contribute to lymphomagenesis under certain circumstances; however, the relationship between a prior rib fracture and lymphoma development remains unclear. In the present case, a direct causal relationship between the previous rib fracture and lymphoma development cannot be established.

This case highlights the importance of considering malignancy in the differential diagnosis of chest wall lesions, even in patients without a history of tuberculosis, chronic pyothorax, or other established risk factors. Nonspecific symptoms, such as chest pain, fever, or general discomfort, should not be overlooked because they may indicate malignant lymphoma. Therefore, early histological examination and appropriate systemic evaluation, including PET-CT, are essential for establishing an accurate diagnosis and initiating timely treatment.

Although systemic chemotherapy and radiotherapy are considered the standard treatment modalities for malignant lymphoma, the role of surgical resection in primary chest wall lymphoma remains controversial. Previous reports have suggested that surgical debulking may be associated with improved survival in selected patients with localized stage I-II DLBCL, particularly when complete or adequate tumor reduction can be achieved [11]. In addition, surgical resection followed by adjuvant chemotherapy has been reported to provide favorable outcomes in selected patients with chest wall lymphoma presenting as a localized mass [12]. Therefore, in cases where the tumor is localized and technically resectable, surgery may contribute not only to establishing a definitive diagnosis but also to improving local control. In the present case, surgical resection was performed because the lesion was solitary and well circumscribed on preoperative imaging, with the aims of establishing a definitive diagnosis and achieving local control.

Once the diagnosis of DLBCL is established, systemic chemotherapy with rituximab, cyclophosphamide, doxorubicin, vincristine, and prednisone (R-CHOP), with or without radiotherapy, is considered the standard treatment strategy [13]. Because our institution does not have a hematology service, definitive treatment could not be initiated on site, and the patient was promptly referred to a tertiary referral center for further management. Institutions without specialized hematology services should similarly ensure timely referral to facilitate the initiation of appropriate treatment without delay.

Nevertheless, as this report describes a single case, the clinical implications regarding the pathogenesis and optimal management of primary chest wall lymphoma should be interpreted with caution. Further accumulation of similar cases is required to clarify the underlying mechanisms and establish optimal treatment strategies.

Primary chest wall lymphoma should be considered in the differential diagnosis of chest wall tumors, even in the absence of chronic pyothorax or tuberculosis. Early tissue diagnosis and appropriate multidisciplinary management may facilitate timely diagnosis and treatment.

## Conclusions

We report a rare case of primary chest wall DLBCL occurring in a patient without a history of tuberculosis or chronic pyothorax. This case highlights that malignant lymphoma should be included in the differential diagnosis of chest wall tumors, regardless of the presence or absence of chronic inflammatory conditions. Prompt tissue diagnosis and comprehensive staging are crucial to avoid delays in appropriate treatment.
